# Supplementary material for: Allosteric competition and inhibition in AMPA receptors
Source: Nat Struct Mol Biol. 2024 Jun 4;31(11):1669–79. doi: 10.1038/s41594-024-01328-0 (PMC11563869; doi:10.1038/s41594-024-01328-0)
Supplement: Supplementary file 1 — Reporting Summary [file 41594_2024_1328_MOESM1_ESM.pdf]

nature portfolio

Corresponding author(s): Edward C. Twomey, Richard L. Huganir, Albert Y. Lau

Last updated by author(s): Apr 23, 2024

Reporting Summary

Nature Portfolio wishes to improve the reproducibility of the work that we publish. This form provides structure for consistency and transparency in reporting. For further information on Nature Portfolio policies, see our Editorial Policies and the Editorial Policy Checklist.

Statistics

For all statistical analyses, confirm that the following items are present in the figure legend, table legend, main text, or Methods section.

n/a	Confirmed	
☐	☒	The exact sample size (*n*) for each experimental group/condition, given as a discrete number and unit of measurement
☐	☒	A statement on whether measurements were taken from distinct samples or whether the same sample was measured repeatedly
☐	☒	The statistical test(s) used AND whether they are one- or two-sided<br>*Only common tests should be described solely by name; describe more complex techniques in the Methods section.*
☒	☐	A description of all covariates tested
☐	☒	A description of any assumptions or corrections, such as tests of normality and adjustment for multiple comparisons
☐	☒	A full description of the statistical parameters including central tendency (e.g. means) or other basic estimates (e.g. regression coefficient) AND variation (e.g. standard deviation) or associated estimates of uncertainty (e.g. confidence intervals)
☐	☒	For null hypothesis testing, the test statistic (e.g. *F*, *t*, *r*) with confidence intervals, effect sizes, degrees of freedom and *P* value noted<br>*Give P values as exact values whenever suitable.*
☒	☐	For Bayesian analysis, information on the choice of priors and Markov chain Monte Carlo settings
☒	☐	For hierarchical and complex designs, identification of the appropriate level for tests and full reporting of outcomes
☒	☐	Estimates of effect sizes (e.g. Cohen's *d*, Pearson's *r*), indicating how they were calculated

Our web collection on statistics for biologists contains articles on many of the points above.

Software and code

Policy information about availability of computer code

Data collection	EPU-3.5, CHARMM36, pCLAMP10
Data analysis	Cryosparc-4.2.1, ChimeraX-1.5, Isolde-1.6, COOT-0.9.8.2, Phenix-1.20, Biopython.pdb, Jalview-2.11.3, Molprobity-4.5.2, OriginPro 2023b, CLampfit-11, SCWRL4, ModLoop Server, CHARMM, T-Coffee, MOLE Online

For manuscripts utilizing custom algorithms or software that are central to the research but not yet described in published literature, software must be made available to editors and reviewers. We strongly encourage code deposition in a community repository (e.g. GitHub). See the Nature Portfolio guidelines for submitting code & software for further information.

Data

Policy information about availability of data

All manuscripts must include a data availability statement. This statement should provide the following information, where applicable:

- Accession codes, unique identifiers, or web links for publicly available datasets
- A description of any restrictions on data availability
- For clinical datasets or third party data, please ensure that the statement adheres to our policy

All cryo-EM reconstructions are deposited into the Electron Microscopy Data Bank (EMDB) and will be released upon publication. The accession codes for GluA2-γ2IS-1 and GluA2-γ2IS-2 are EMDB-43275 and EMDB-43276, respectively. The full maps (prior to local refinements and signal subtraction) are the primary cryo-EM maps in each deposition and each local map are supplied as supplemental files in each deposition. The GluA2-γ2IS-1 and GluA2-γ2IS-2 are deposited in the Protein

Data Bank and will be released upon publication. The PDB access codes for GluA2-γ2IS-1 and GluA2-γ2IS-2 are 8VJ6 and 8VJ7, respectively. All conformers from MD simulation trajectories, data from umbrella sampling, analysis code, will be publicly available from Zenodo upon publication of this work accession #10967297. DOI: 10.5281/zenodo.1096729

Research involving human participants, their data, or biological material

Policy information about studies with human participants or human data. See also policy information about sex, gender (identity/presentation), and sexual orientation and race, ethnicity and racism.

Reporting on sex and gender	N/A
Reporting on race, ethnicity, or other socially relevant groupings	N/A
Population characteristics	N/A
Recruitment	N/A
Ethics oversight	N/A

Note that full information on the approval of the study protocol must also be provided in the manuscript.

Field-specific reporting

Please select the one below that is the best fit for your research. If you are not sure, read the appropriate sections before making your selection.

☒ Life sciences ☐ Behavioural & social sciences ☐ Ecological, evolutionary & environmental sciences

For a reference copy of the document with all sections, see nature.com/documents/nr-reporting-summary-flat.pdf

Life sciences study design

All studies must disclose on these points even when the disclosure is negative.

Sample size	Sample size was not predetermined prior to study, but was determined by the availability of microscope time. For smFRET, sample size was dictated by molecules with a single photobleaching event. This stringent criterion ensured that only one donor and one acceptor fluorophore were attached to each GluA2 protein. For electrophysiology of our cryo-EM construct, we predetermined to record each condition in triplicate in lieu of statistic-based sample size determination. Samples were patched if they fluoresced green from the cryo-EM construct.
Data exclusions	No data was excluded.
Replication	Image processing in cryo-EM was duplicated and performed with ab initio models generated from the data. No external data was input into the image processing. All successful electrophysiological recordings were reproducible. Dose response recordings for allosteric competition were repeated in triplicate. Sweeps per cell are also reported. For smFRET, replaces were: GluA2-γ2FRET (CTZ = 76, GYKI-52466 = 77), GluA2FRET (CTZ = 62*, GYKI-52466 = 96). 30 molecules with 1 mM of glutamate and 100 µM CTZ were obtained from Carrillo and Shaikh et al (2020). All attempts to reproduce experimental data were successful.
Randomization	These experiments were not randomized. Covariates were minimized by comparing different conditions on the same experimental day to minimize batch effects.
Blinding	The investigators were not blinded to the data analysis. This is not technically or practically feasible for Cryo-EM, patch clamp, smFRET, or Molecular Dynamics studies. Researchers conducting the data analysis for each experiment were also responsible for data collection, making blinding impossible.

Reporting for specific materials, systems and methods

We require information from authors about some types of materials, experimental systems and methods used in many studies. Here, indicate whether each material, system or method listed is relevant to your study. If you are not sure if a list item applies to your research, read the appropriate section before selecting a response.

Materials & experimental systems

n/a	Involved in the study
☐	☒ Antibodies
☐	☒ Eukaryotic cell lines
☒	☐ Palaeontology and archaeology
☒	☐ Animals and other organisms
☒	☐ Clinical data
☒	☐ Dual use research of concern
☒	☐ Plants

Methods

n/a	Involved in the study
☒	☐ ChIP-seq
☒	☐ Flow cytometry
☒	☐ MRI-based neuroimaging

Antibodies

Antibodies used

biotinylated Goat Anti-Mouse IgG (H + L) secondary antibody (Jackson Immunoresearch Laboratories, catalog number 115-065-003) used at 2.7ng/ul
anti-GluR2 , Clone: L21/32(BioLegend®) - used at 3.0ng/ul
anti-TARPγ2, Clone: N245/36 (Millipore) - used at 2.4ng/ul

Validation

biotinylated Goat Anti-Mouse IgG (H + L) secondary antibody - From manufacturer: Based on immunoelectrophoresis and/or ELISA, the antibody reacts with whole molecule mouse IgG. It also
reacts with the light chains of other mouse immunoglobulins. No antibody was detected against non-immunoglobulin serum proteins. The antibody may cross-react with immunoglobulins from other species.

anti-GluR2 - From manufacturer: Cross-reacts with Human, Mouse, Rat; Each lot of this antibody is quality control tested by Western blotting; It does not cross-react with GluA1/GluR1, GluA3/GluR3, or GluA4/GluR4.; References: 1. Brown EA, et al. 2018. Mol Autism. 9:48. 2. Lautz JD, et al. 2021. Cell Rep. 37:110076.

anti-TARPγ2- From manufacturer:Each new lot of antibody is quality control tested by western blot on rat whole brain lysate and confirmed to stain the expected molecular weight band.

Eukaryotic cell lines

Policy information about cell lines and Sex and Gender in Research

Cell line source(s)

Sf9 for baculorvirus (Gibco, A35243); Expi293 Gnti- for protein overexpression (Gibco, A39240). HEK293T (ATCC, CRL-3216) for smFRET and electrophysiology.

Authentication

Sf9s cells are routinely used in our labs and were not specifically validated for these studies outside of the manufacturer's specifications. Expi293 Gnti- cells are routinely used in our labs and were not specifically validated for these studies outside of the manufacturer's specifications.

Mycoplasma contamination

Sf9 cell lines tested negative for mycoplasma. Expi293 Gnti- cell lines tested negative for mycoplasma. HEK293T tested negative for mycoplasma.

Commonly misidentified lines
(See ICLAC register)

No commonly misidentified lines were used for this study.

Plants

Seed stocks

N/A

Novel plant genotypes

N/A

Authentication

N/A
