# Supplementary figures and images for: Allosteric competition and inhibition in AMPA receptors
Source: Nat Struct Mol Biol. 2024 Jun 4;31(11):1669–79. doi: 10.1038/s41594-024-01328-0 (PMC11563869; doi:10.1038/s41594-024-01328-0)

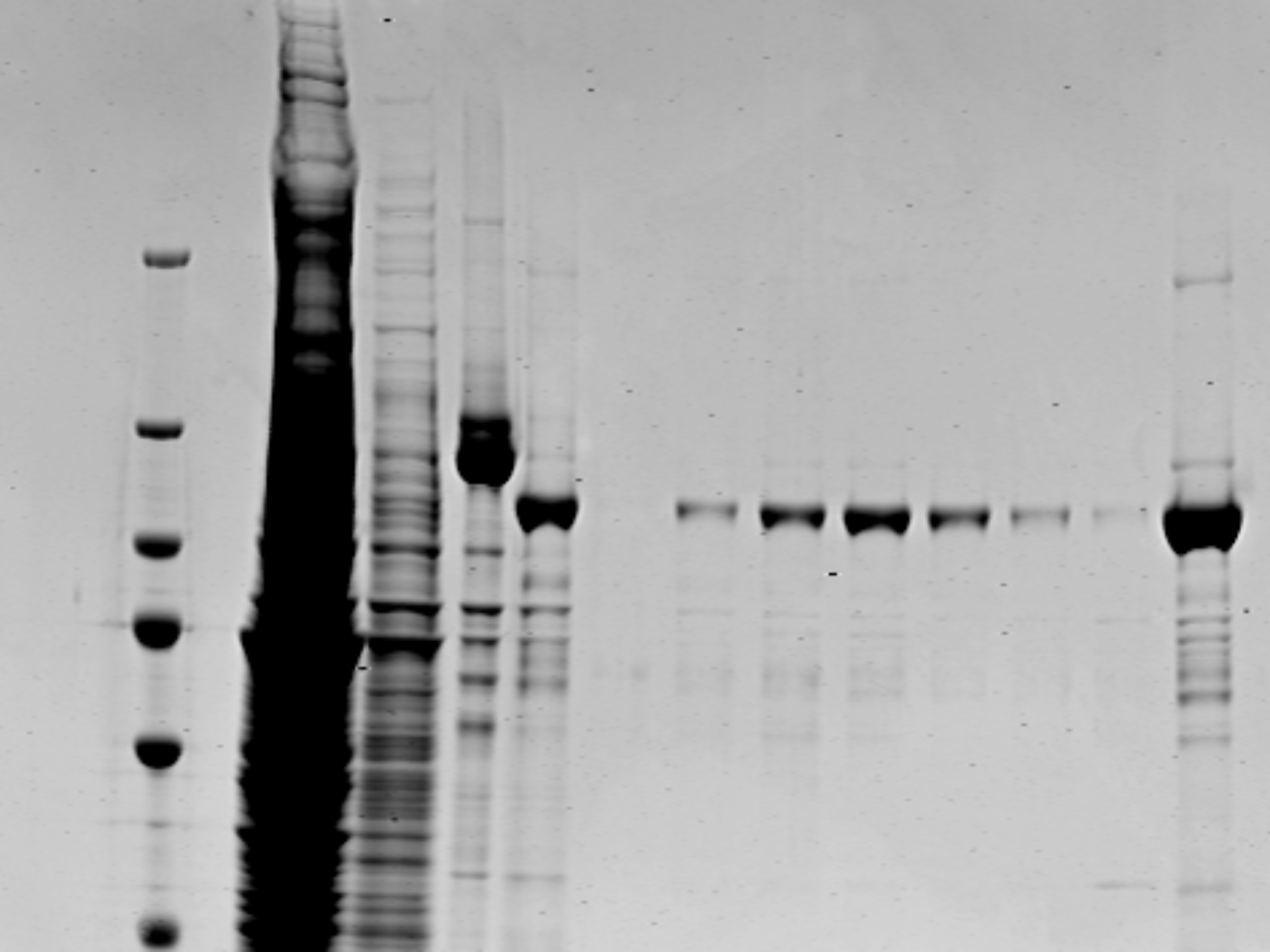

Supplement: Supplementary file 8 — Full uncropped gel corresponding to Extended Data Fig. 1c. [file 41594_2024_1328_MOESM8_ESM.tif]
